# Miniterm, a Novel Virtual Sensor for Predictive Maintenance for the Industry 4.0 Era

**DOI:** 10.3390/s22166222

**Published:** 2022-08-19

**Authors:** Eduardo Garcia, Nicolás Montés, Javier Llopis, Antonio Lacasa

**Affiliations:** 1 Ford Spain, Poligono Industrial Ford S/N, 46440 Valencia, Almussafes, Spain; 2 Department of Mathematics, Physics and Technological Sciences, University CEU Cardenal Herrera, C/ San Bartolome 55, 46115 Valencia, Alfara del Patriarca, Spain

**Keywords:** Miniterm, IIoT, Industry 4.0, fault detection, sub-cicle time, virtual sensor

## Abstract

This article introduces a novel virtual sensor for predictive maintenance called mini-term. A mini-term can be defined as the time it takes for a part of the machine to do its job. Being a technical sub-cycle time, its function has been linked to production. However, when a machine or component gets deteriorated, the mini-term also suffers deterioration, allowing it to be a multifunctional indicator for the prediction of machine failures as well as measurement of production. Currently, in Industry 4.0, one of the handicaps is Big Data and Data Analysis. However, in the case of predictive maintenance, the need to install sensors in the machines means that when the proposed scientific solutions reach the industry, they cannot be carried out massively due to the high cost this entails. The advantage introduced by the mini-term is that it can be implemented in an easy and simple way in pre-installed systems since you only need to program a timer in the PLC or PC that controls the line/machine in the production line, allowing, according to the authors’ knowledge, to build industrial Big Data on predictive maintenance for the first time, which is called Miniterm 4.0. This article shows evidence of the important improvements generated by the use of Miniterm 4.0 in a factory. At the end of the paper we show the evolution of TAV (Technical availability), Mean Time To Repair (MTTR), EM (Number of Work order (Emergency Orders/line Stop)) and OM (Labour hours in EM) showing a very important improvement as the number of mini-terms was increased and the Miniterm 4.0 system became more reliable. In particular, TAV is increased by 15%, OM is reduced in 5000 orders, MTTR is reduced in 2 h and there are produced 3000 orders less than when mini-terms did not exist. At the end of the article we discuss the benefits and limitations of the mini-terms and we show the conclusions and future works.

## 1. Introduction

A production, manufacturing or assembly line can be defined as a group of sequential operations established in a factory where the product is moved through them while the final product is built. The design of these types of lines is the first key factor, there are a large number of crucial decisions that will be made during product design, line format configuration, line balancing, machine selection, technology available, etc. One of the most critical parameters when designing a line is the cycle time. This is defined as the time the part takes to be made. In a “perfectly” balanced line, it is the time spent on each position of the line to manufacture each of the parts for the final product. In general, these problems are considered one at a time, due to their complexity.In general, these problems are considered one at a time, due to their complexity. Hence, the great importance of the line balancing where the tasks are assigned to the work stations and taking into account the resources used. Due to the importance of this task, a large number of researchers have been working on this topic. Under the acronym ALB (Assembly Line Balancing), a large number of optimization models have been presented and discussed in scientific and technical literature [1].

### 1.1. What Happens after the Installation of the Production Lines?

Once the line has been installed and begins to produce parts, this comes under the control of the factory management. They need to find a technically feasible point in which maintenance and product quality levels should be selected to achieve the highest productivity in order to meet the company’s objectives for greater profitability. The correct combination of three factors, production, quality and maintenance, is the key to competitiveness of the company.

***Quality and production***: The link between quality and production is perfectly defined in the literature [2,3]. The improvement of the quality implies eliminating, for example, imperfections that may cause rework, which would increase the production cost and, consequently, the final cost of the part.

***Maintenance and production***: Maintenance is a function of the factory management that runs in parallel with production. The main output of the production is the expected product and its secondary output is a maintenance demand, which is the input of the maintenance function. The result of maintenance is an additional production input considered as *production capacity*. While production manufactures the product, maintenance provides the production capacity. Thus, maintenance affects production by increasing its capacity and controlling the quantity and quality of the output (the product) [2].

***Maintenance and quality***: The role of maintenance in the long-term use of manufacturing lines is well known and taken into account by researchers and engineers. There are many strategies that seek to increase the long service life of the lines. However, extending the useful life of the line is pointless without quality criteria being maintained [2]. In general terms, the equipment that is not properly maintained has periodic failures, suffers speed losses and loses precision and therefore tends to cause faults [2]. On the other hand, excessive maintenance may result in unnecessary costs.

***Maintenance, quality and production***: Determining the optimal time in which to perform the maintenance task is the key to be able to link the three factors. In [4] an analytical study has been conducted for a food products company to see the link between these three factors. The link between maintenance and production is positive. This implies that, the more maintenance, the machines work in better conditions, generating a continuous production. The link between quality and production is negative. This implies that the quality control activities expose production. Lower production is obtained the more hours are invested in quality control.

#### 1.1.1. Production

Focusing our attention on production, the line is designed by a team of experts, based on all the parameters, also defining the maximum capacity of production. In the automotive sector, this maximum capacity is measured by the JPH (jobs per hour) and it is known as “Engineering Running Capacity” (ERC). The objective of those responsible for production is to ensure that the line will reach this maximum value. The reality of the manufacturing lines shows that the ERC cannot be reached in practice, so the production engineers have estimated a new maximum production rate which is known as "Engineering Running Rate" (ERR). This new rate can be defined empirically, based on observations of the line performance. This mismatch between ERC and ERR can be due to a multitude of factors since, during the useful life of a production line, which can be decades, performance depends on a large number of parameters such as maintenance, stopping time events, equipment breakdowns, waiting systems, the dynamic behaviour of bottlenecks, the Bowl phenomenon, market demand, new available technologies, etc. In fact, each workstation has its own identity as they are not intrinsically identical [5]. This means that many of the simplified versions of the models or line balancing algorithms are not applicable to real lines, having to be adapted “manually” [5,6].

#### 1.1.2. Maintenance

Another key factor in the performance of the manufacturing lines is maintenance. In general, maintenance can be classified as two main groups: Corrective Maintenance (CM) and Preventive/Predictive maintenance (PM). CM is carried out when the machine fails or some the equipment elements are damaged and must be replaced or repaired, this element and/or part will be responsible for a failure in the whole line if the action is not implemented. However, the PM is carried out before the equipment fails. The purpose of a PM order is to promote continuous production of the system and/or minimize the loss of performance. In the preventive/predictive maintenance, we can find two great types of strategies: based on time (Time-based Maintenance, TBM) or those based on the state of the machine (Condition Based Maintenance, CBM). Those strategies based on time involve carrying out a preventive maintenance periodically, lubricating, calibrating and performing periodic controls. Instead, the CBM strategy involves making a real-time diagnosis where the decision is made by observing the “condition” of the system and its components [6]. TBM strategies are based on the manufacturer’s recommendations, fault history, operators and/or maintenance staff’s experience. In contrast, in the CBM strategy, the objective is avoiding unnecessary maintenance tasks and performing them when there is evidence of abnormal functioning. It is a proactive strategy in which the development of a predictive model is required. The CBM is that 99 % of equipment failures are preceded by certain signs, conditions or indications that the failure is about to occur [7]. The system condition is quantified through sensor measurements taken periodically and even continuously [6,8]. In general, the purpose of the CBM is twofold. First, collecting data on the machine condition and second, increasing the knowledge of the causes for the failures, the effects and the deterioration patterns of the equipment [8]. In addition, CBM, through this strategy, can ensure a high quality of the final product, especially if the thresholds of the measurements being taken from the machine are correctly selected [2]. CBM can be carried out in two ways: online or offline [8]. The online process involves carrying it out while the machines are active. On the contrary, in offline mode, the process is performed while the machine is stopped. In this case we usually look for cracks, colour changes, etc. Moreover, the CBM can be done continuously or periodically. The most usual way is to do it periodically, for example, every hour or every change of shift, although the ideal way would be to do it continuously and automatically. However, as indicated in [9], it may be very expensive since many sensors and devices are needed to carry it out. The most commonly used sensors to perform the CBM are the following:

- *Vibration*: Vibration sensorization is one of the most commonly used techniques for CBM, especially for machines with rotating elements [10]. The analysis is done in situ and it is a non-destructive test.
- *Noise*: It is another of the most used techniques in CBM. It has a strong link with vibration and therefore it is also used for machines with rotating elements [10]. However, there is a fundamental difference between the two. While the sensorization of the vibration requires being in contact with the machine or element to be sensorized, noise monitoring is simply “listening” to the equipment without having to be in contact [8].
- *Analysis of the oil or lubricant*: With this technique, the oil is analysed to determine whether it is able to function or not properly. In addition, it also provides an indirect measure of the deterioration level of the components lubricated [8].
- *Electrical measurements*:This technique includes the measurement of changes in the properties of equipment such as resistance, conductivity and insulation. This technique is usually carried out to detect deterioration of insulation in engines,
- *Temperature*: This technique is used primarily to detect faults in electrical and electronic components [8].
- *Pressure, flow, electric consumption*: These techniques are also used, although to a lesser extent than the previous ones.

The decisions to be made under the CBM concept can be classified into two: Diagnosis and prediction. Diagnosis is the process of finding the source of the failure while prediction is the process of estimating when the failure will occur [11]. The objective of the diagnosis is to warn maintenance engineers on equipment operations under abnormal functioning conditions. Even if the equipment is working in abnormal conditions, this does not mean the equipment has failed. This will happen after a certain time [8]. The time that remains until the failure happens is the one that must estimate the prediction. Regarding maintenance, prediction is much more relevant than the diagnosis since unexpected failures can be predicted [9].

#### 1.1.3. Change Point Definition

**Figure 1:**
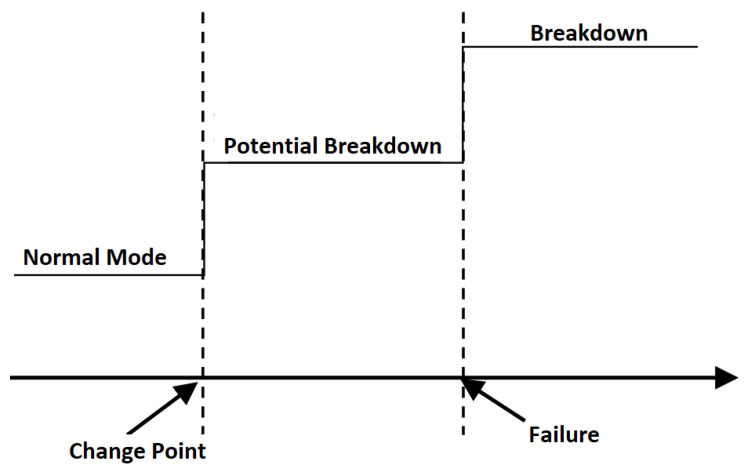
Change point definition.

At this point, what is known as “Change point” comes into play, see Figure 1.

The change point is defined as an abrupt change in the time series measurement that is being made of the machine, vibration, noise, etc. More formally, let us assume we have an ordered sequence of data, $y_{1:n}=\left(y_{1},\ldots ,y_{n}\right)$. A changepoint is said to occur within this set when there exists a time, t∈{1,…,n−1}, such that the statistical properties of $\left\{y_{1},\ldots ,y_{t}\right\}$ and $\left\{y_{t+1},\ldots ,y_{n}\right\}$ are different in some way.

**Figure 2:**
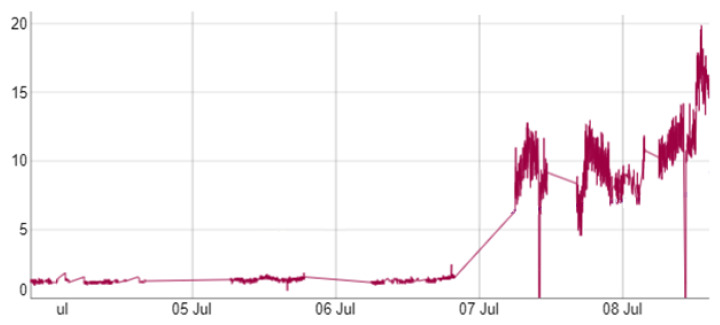
Change point of an elevator measured with a vibration sensor at the Ford factory located in Almussafes (Valencia).

The change point is an indication that something anomalous is happening and announces the end of the useful life of some component. In [12] an attempt is made to define a guide on how to deal with CBM. As an example, Figure 2 shows the deterioration of an elevator measured with a vibration sensor at the Ford factory located in Almussafes (Valencia).

The change point is always related with some physical change of the component. In the case of oil or lubricant, we know there is a sudden change in performance, mainly because, when the oil is approaching the end of its useful life, its viscosity changes abruptly. When a component or part is subjected to a constant load, the elongation that suffers over time is known as “the creep curve” where, at the end of its life there is an accelerated elongation, see [13]. Something similar happens with the elasticity coefficient. When a part is subjected to continuous bending, such as a train track, see [14,15], and the end of its useful life is approaching, there comes a point where it will not recover its starting position. There are different techniques to detect change points, such as EWMA, CUMSUM, MSE, etc., see [15]. Given their relevance to CBM, new techniques are being researched for more complex cases, see [16], and a special package has even been developed for R, see [17].

## 2. Previous Results by the Research Team

In [18] an improvement of the existing mathematical model in the literature on production lines was proposed, and its use in the improvement of the manufacturing process. The literature classifies the time data used in the analysis of the manufacturing process into two types, the long-term data (long-terms), and the short-term data (short-terms). Long-term data time are used mainly for process planning while short-term data time are used mainly for process control. There is abundant literature in which the analysis of long-term times is studied, in comparison with the literature that studies short-term times. Following the definition by [19], the short-term data refer to a time not long enough for the failure period of the machine and where the cycle time of the machine is considered short-term time.

### 2.1. From Long-Term to Micro-Term Cycle Time Data Model

**Figure 3:**
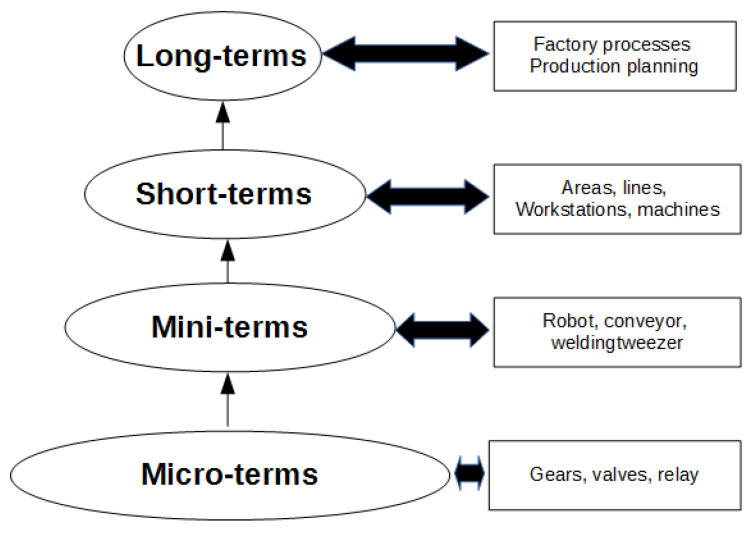
From the micro-term to the long-term.

In [18], the short-term is redefined in two new terms: the mini-term data time and the micro-term data time. A mini-term can be defined as the time it takes for a part of the machine to do its job. The division of a machine into mini-terms can be conditioned by, in a preventive maintenance policy or in a breakdown, the component that could be replaced in an easier and faster way than another subdivided part of the machine. A mini-term could also be defined as a sub-division that allows us to understand and study the machine behaviour. In the same way, a micro-term is defined as each part of the mini-term that could be divided into itself, see Figure 3. A mini-term can be calculated by adding the micro-terms into which the mini-term is divided. In the same way, a short-term can be calculated adding the mini-terms into which the short-term is divided. The same with long-term and short-term. For more information about this multi-scale time analysis in dynamical systems, please check our previous works [18,20,21].

The mathematical model proposed in [18] was reformulated in [20], using tensor algebra, which reduces the computational cost of the model, particularly when the number of mini-terms and micro-terms is high.

More recently, in [22] the data model is mixed with the complete modeling of a factory using Petri nets to develop a manufacturing map, a hierarchical construction of Petri nets in which the lowest level network is a temporary Petri net based on mini-terms, and in which the highest level is a global view of the entire plant. The user of a manufacturing map can select intermediate levels, such as a specific production line, and perform analysis or simulation using real-time data from the mini-term database.

### 2.2. The Mini-Term: The Link between Production and Maintenance

One of the contributions made in [18] was the use of the mini-term to link production and maintenance. The mini-term, by definition is a sub-cycle time and it would only make sense to use it for production improvement. More formally, the mini-term can be defined as an ordered sequence of sub-cycle times, $m_{1:n}=\left(m_{1},\ldots ,m_{n}\right)$.

**Figure 4:**
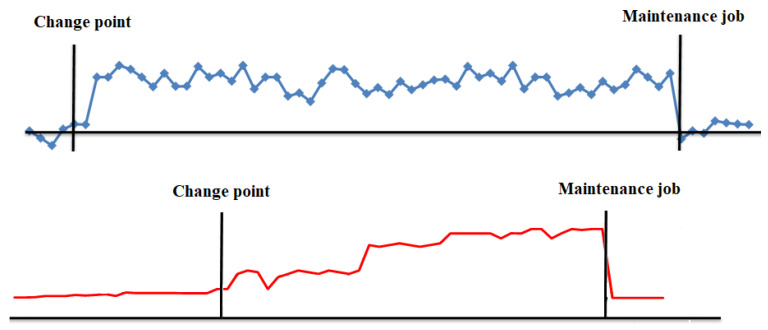
Change points in mini-terms. Proportional valve (**above**). Leak in cylinder (**below**).

However, in [18] it is shown that when a deterioration happens and ends up becoming a change point in vibration sensors (see Figure 2), this change point also happens in the mini-term, that is, a physical change point will result in a deterioration of the cycle time. More formally, a change point in the mini-term is said to occur within this set when there exists a time, t∈{1,…,n−1}, such that the statistical properties of $\left\{m_{1},\ldots ,m_{t}\right\}$ and $\left\{y_{m+1},\ldots {,}_{n}\right\}$ are different in some way. Figure 4 shows two examples of change points in mini-terms of a welding clamp measured at Ford factory in Almussafes. The first is due to the deterioration of the proportional valve that controls the clamp movement. The second is due to an internal leak in the clamp cylinder.

**Figure 5:**
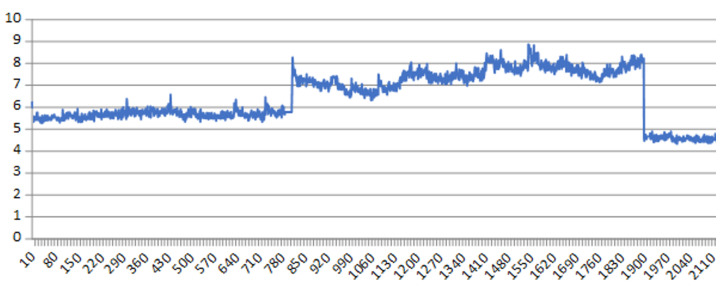
Change points in mini-terms of a welding clamp due to lack of lubrication.

In the mini-term we can see reflected all the physical change points that take place in the machine, and these change points result in a deterioration of the sub-cycle time. The example in Figure 5 shows the effect on the mini-term that the deterioration of the lubricant in the welding clamp has and how, once lubricated correctly, it recovers its nominal value. It means that mini-term colud act as a “virtual” sensor for predictive maintenance tasks.

### 2.3. Benefits Using Mini-Term in the Industry 4.0 Era

One of the main handicaps of Industry 4.0 is the cost of adding sensors in the machines and how to integrate them with the installed systems. As explained in the previous section, there are different prediction systems based on vibration, noise, temperature sensors, etc., but they are excessively expensive if we think about using them in a massive way for all the machines/components that we can find in a car factory, for example. It would take a large number of sensors, wiring the installation, programming the measurement, etc., and for this reason, these techniques are not used in a massive way, only for critical machines. Thus, the success or failure of the proposals related to IIOT (Industrial Internet of Things) and Industry 4.0 is mainly influenced by the cost of the proposals, the number of devices to connect and their interaction with the pre-installed systems in the production process.

However, mini-terms (technical sub-cycle times) could be measure with the PC/PLC sensors and the industrial network already installed in the production line which is responsible for the automatic production of the lines, doing the installation process, cheap and easy to install. It allows, for the first time, to create a IIOT Big Data based on Miniterms to predict failures in production lines

### 2.4. Installation Setup

As mentioned above, the objective is to measure the mini-terms (technical sub-cycle times) with the PC/PLC sensors and the industrial network already installed in the production line which is responsible for the automatic production of the lines. To do this, the first step was to include the mini-terms in Ford’s standards in order to begin mass programming. In large companies like Ford, there are standards and protocols used to program PLCs, with I/O restrictions, memory, etc. No supplier can program a PLC if he/she does not know the standard.

Thanks to standardization, it is currently possible to program a mini-term in any PLC that Ford has in any factory in the world where the industrial network is in charge of channeling the mini-terms to the Database. With this it is possible to measure the mini-term, with the only cost of programming a timer in the PLC/PC allowing, for the first time, the massive monitoring of the time it takes for the components of the machines to carry out their task.

**Figure 6:**
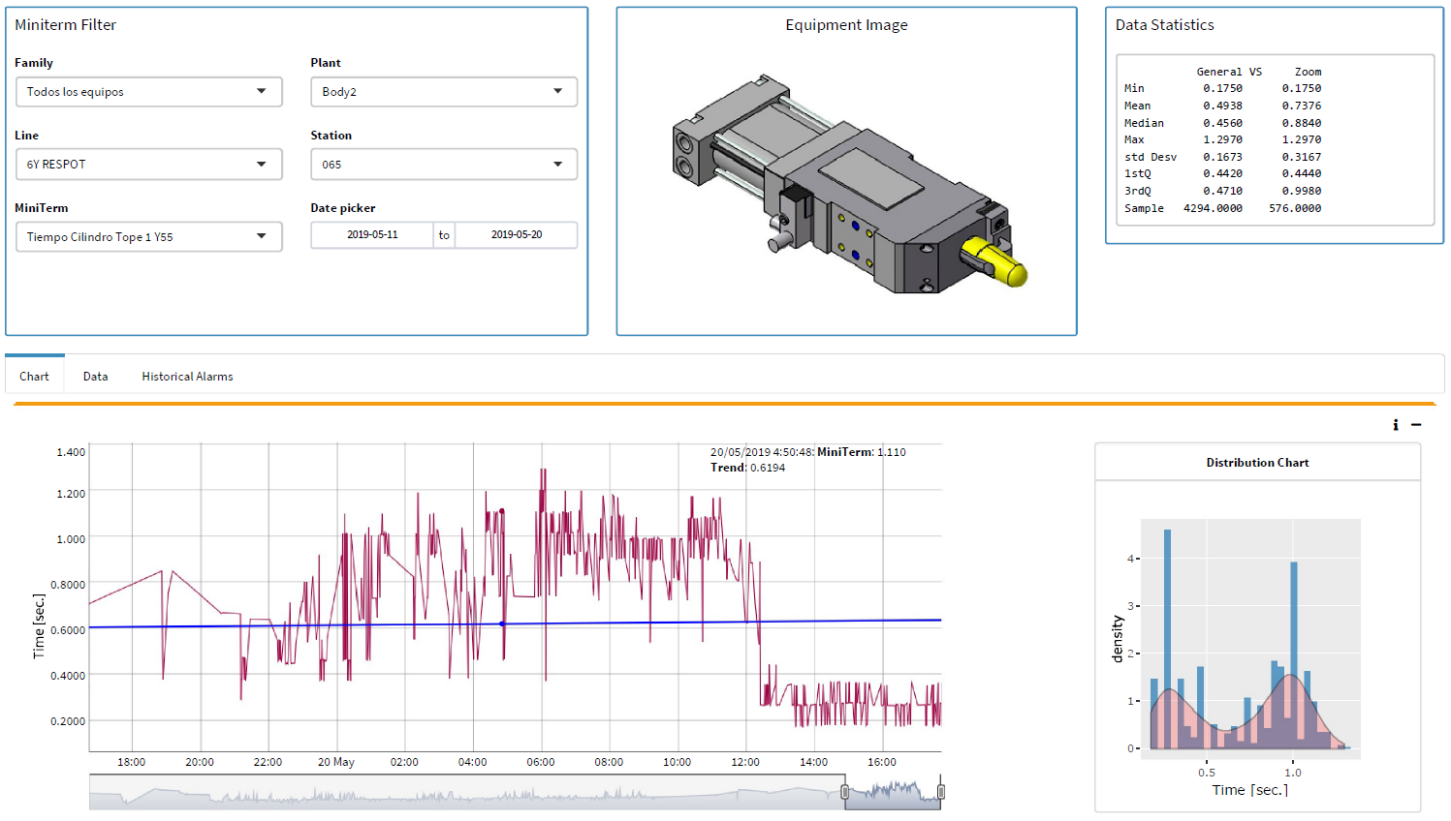
Architecture for Miniterm 4.0. It collects Miniterms in Real-time at Ford factories.

In order to activate and monitor the mini-terms and implement the project, a software interface programmed in R was developed, see Figure 6.

## 3. Objective of Our Line of Research

**Figure 7:**
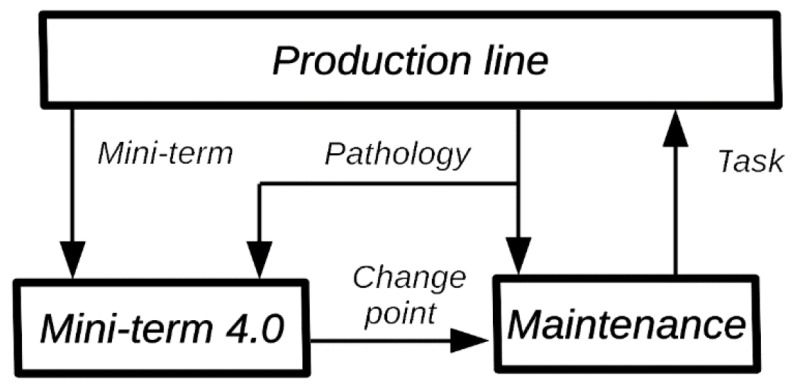
Miniterm 4.0 architecture to predict failures through mini-terms.

The objective of our line of research is to create the intelligent system “Miniterm 4.0” where, through real-time monitoring of the mini-terms, we will be able to predict the failures of the monitored components, determine their pathology and determine the effect they will have on production and the quality of the manufactured part, see Figure 7. This article shows the results for the first part of the goal: predict the failures. During the last years, Ford factory in Almussafes (Valencia) has been programming mini-terms in different components like cylinders, clamps, robots, elevators, conveyors, gearbox, fans, switches, turntables, etc. Nowadays, the number of mini-terms are up to 22 K. During the installation process, the availability indicators of the machines and production have improved significantly as the mini-terms have been installed and the detection algorithms have been improved. Section 4 shows the evolution and current algorithm for detecting change points by using mini-terms, the common causes that show false positives and how they are being solved. Section 5 shows the results and goodness of the system from the point of view of the improvement in the production indicators, TAV, OM EM, etc. Section 6 shows the conclusions and future work.

## 4. Towards Robust Detection of the “Change Point” of Mini-Term

**Figure 8:**
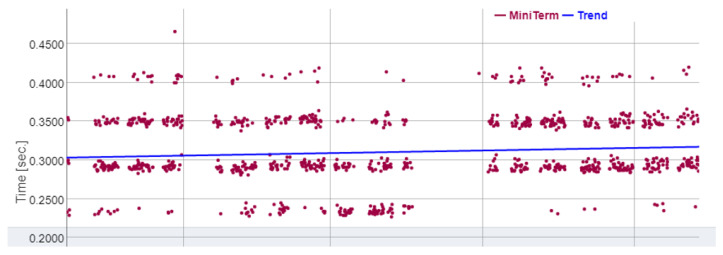
Change point produced by Scan-Time.

The mini-terms installation process started in mid-2018. In the first version of the application, a basic state change detection algorithm was implemented to detect change points that would allow us to automatically detect when a mini-term had changed its behaviour significantly. The algorithm implemented was a K-Means classification algorithm [21]. This algorithm divided the data accumulated in the previous 9 days into two groups, based on the mean value of the groups. Two thresholds were established. A 7% and an 18%, the first one was used to set up an alarm configuring that component in warning mode and the second to automatically send an e-mail to the maintenance workers so that they could proceed to check the component [21]. This algorithm allowed us to store time series with change points that took place in the plant. After several months accumulating in the database the real cases detected, we were able to get an idea of the different types of change points that could exist. The 10 most representative ones were selected and all the change point algorithms programmed in R were tested [23]. A comparison was made, both in its effectiveness and precision in detecting the change point, as well as in its computation time. From this comparison, the result showed that Bartlett’s algorithm was the most effective one in which the version of the winding sliding algorithm is the most efficient at computational level [24]. This algorithm was implemented in the system, replacing the previous one. This new version of the algorithm made it possible to replace the change point based on a percentage of the mean by a *p*-value, the percentage of success showing that a change point has actually taken place, as well as its location in the time series. These two improvements led to an improvement in detection, but there were also new false positives and negatives caused by:

- ***Oscillatory change point***: There are pathologies/components that, when an anomaly occurs, this results in a fluctuation in the mini-term but it does not result in a change in the mean value of the data, making it undetectable by Bartlett’s algorithm, causing false negatives.
- ***Slow deterioration***: There are pathologies that generate a slow deterioration where the mini-term increases its value progressively but slowly and it is necessary to compare it with previous months to determine the deterioration caused. False negatives.
- ***Scan-Time (PLC’s sampling frequency)***: One of the handicaps that have come up due to the massive use of mini-terms is the Scan-Time. Scan-Time is the time it takes for the PLC to collect the inputs, execute its PLC program and then update the outputs. Since the objective is to use the PLC’s already installed to measure the mini-terms, it is a parameter that is imposed and generates false positives. Figure 8 shows the effect produced by the Scan-Time on the measured data. This effect causes false positives when the Scan-Time is high in relation to the mini-term value.

## 5. Current Mini-Term Anomaly Detection Algorithm

In the literature, an initial calibration is usually carried out to adjust the algorithms to the initial state of the machines or components, see [25]. On the other hand, there is a widespread use of numerical indicators in the detection of change points in the industry, see [26,27,28]. The great advantage of using numerical indicators is the possibility of adapting and developing new indicators in order to adapt them to different types of characteristics. However, this advantage could be a problem if you want to generalize the indicators for whatever component, that is the case of mini-terms. Therefore, a robust and general algorithm is welcome for those cases.

Most common indicators, see [26,27,28], Mean, variance, Skewness, Kurtosis, etc., have the same initial supposition, that is, the data you collect for a machine with good health follows a normal distribution. In statistics, when you have a set of data that follows a normal distribution, the methods to detect outliers are well known. In statistics, an outlier is a data point that differs significantly from other observations.These values are commonly excluded from the data set because can cause serious problems in statistical analyses. There are some methods and techniques to determine outliers such as Peirce’s criterion or Thompson Tau and Modified Thompson Tau test. Other methods based on observations based are based on measures such as the interquartile range. For example, if $Q_{1}$ and $Q_{3}$ are the lower and upper quartiles, respectively, then we could define an outlier as an observation outside the range:

(1) $$[Q_{1}-k\left(Q_{3}-Q_{1}\right),Q_{3}+k\left(Q_{3}-Q_{1}\right)],$$

for some nonnegative constant k. John Tukey proposed this test, where k=1.5 indicates an “outlier”, and k=3 indicates data that is “far out”.

Currently, outlier detection using the previous formula is used to determine if the mini-term is not working properly. The procedure to use is as follows:

- When a mini-term is registered, an initial calibration is carried out using an initial set of mini-terms $\left\{m_{1},\ldots ,m_{t}\right\}$ to determine how the machine or component performs in normal operation by adjusting the interquartile range. During the registration process, the operator has a chart of the data to decide if it follows a normal distribution. If two overlapping normal distributions appear in that chart, the mini-term would be considered as “programming error”.
- The limits of the alarms are defined with the calculation of the quartiles using the initial set of mini-terms $\left\{m_{1},\ldots ,m_{t}\right\}$. If the anomaly is in the range k=[1.5−3] it is defined as a *Warning* while if the anomaly is in the range >3 the alarm is defined as type *Red*.
- If an alarm occurs and it is classified as *Warning*, an e-mail is sent to the head of maintenance, who decides if the variation is considered sufficient to be sent to the maintenance operators.
- If an alarm occurs and it is classified as *Red*, an e-mail is sent directly to the maintenance operators who will check the component.

**Figure 9:**
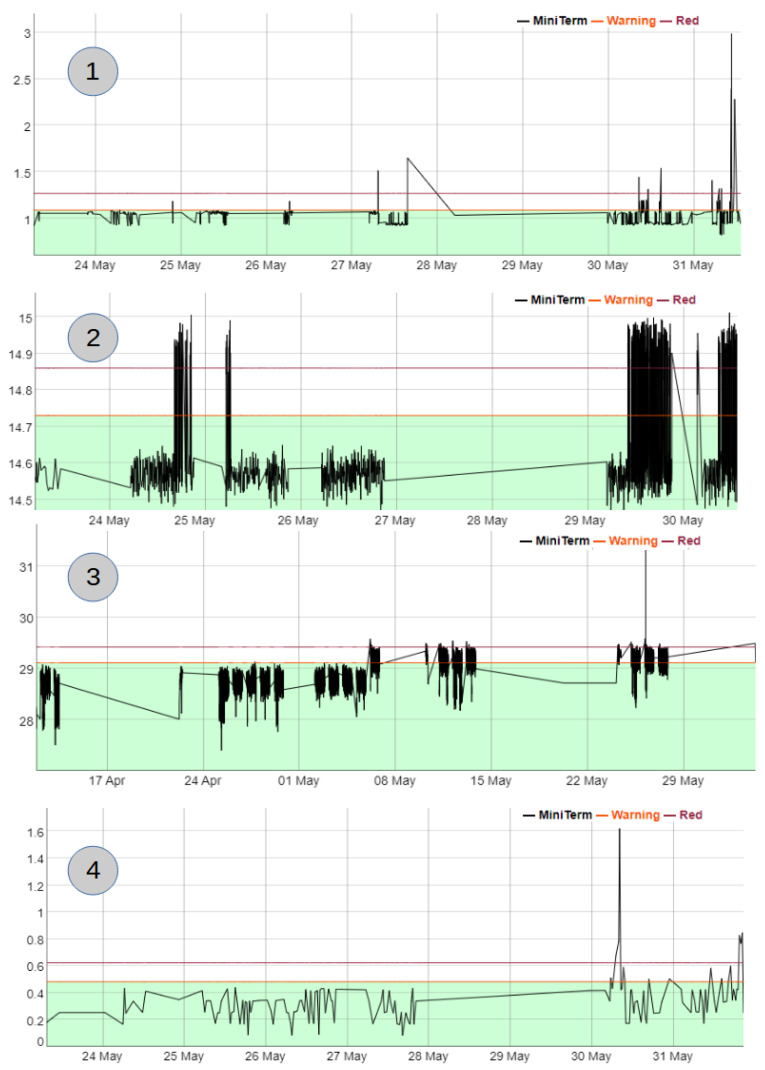
Examples of mini-term alarm. (**1**) Clamp, (**2**) Rollertable, (**3**) Lifter, (**4**) Welding gun.

Figure 9 shows some examples of mini-terms where 1 is an alarm that happened in a clamp, 2 is an alarm in a rollertable, 3 is in a lifter and 4 in a welding gun.

## 6. Experimental Results

The process of installation and monitoring of machines and components began in 2018, in which 200 mini-terms were installed. In 2019, we were granted the project funded by the CDIT (IDI-20190878) (Centre for the Development of Industrial Technology), a Public Business Entity, under the Ministry of Science and Innovation which promotes innovation and technological development for Spanish companies. This project made it possible to massively implement the mini-terms at the Ford Factory in Almussafes (Valencia) and develop the necessary algorithms for the detection of machine failures. To date, June 2022, there are 18,893 mini-terms installed, of which 15,009 are pneumatic cylinders, 427 are pneumatic welding clamps, 24 pumps, 131 pantographs, 216 fans, 123 anchor matrices, 597 servos, 1690 tables and 479 rotary tables, 161 switches and 36 reducers.

### 6.1. Effectiveness of the Detection Algorithm

As explained in the previous section, the fault detection algorithm has undergone different evolutions. Initially, an algorithm based on K-Means was developed [21], which made it possible to collect cases of faults and carry out a more in-depth study of change point detection algorithms, see [24], where the conclusion was that Bartlett’s algorithm was the most suitable one. However, this algorithm still had problems when the anomaly was fluctuating, or slow, and also detected the Scan-Time as an anomaly. To solve it, in this article an algorithm based on outlier detection from an initial calibration has been proposed. Although the proposed algorithm is very simple, it is extremely robust because in the initial calibration process, it is able to absorb the Scan-Time of the mini-term and it will not be detected as an anomaly. In addition, the slow deterioration can be detected as it starts from an initial calibration which can find any slow deterioration. The same is applied to oscillation pathologies. Thus, the effectiveness limit mentioned in [7] has been reached, where it was indicated that 99% of equipment failures are preceded by certain signs, conditions or indications that the failure is about to occur. That 1% remaining corresponds to components that have a sudden failure.

### 6.2. Benefits of Using Mini-Terms in Industry

Since the mini-terms began to be installed and used to predict breakdowns, different types of indicators have begun to undergo significant improvements, including:

- Technical availability (TAV): Percentage of planned production time without unexpected technical difficulties or maintenance needs.
- Mean Time To Repair (MTTR): Average time required to repair a failed component or device.
- Mean Time Between Failure (MTBF): Elapsed time between inherent failures of a mechanical or electronic system, during normal system operation.
- Number of Work order EM (Emergency Orders/line Stop).
- Labor hours in EM (Emergency Orders/line Stop).

**Figure 10:**
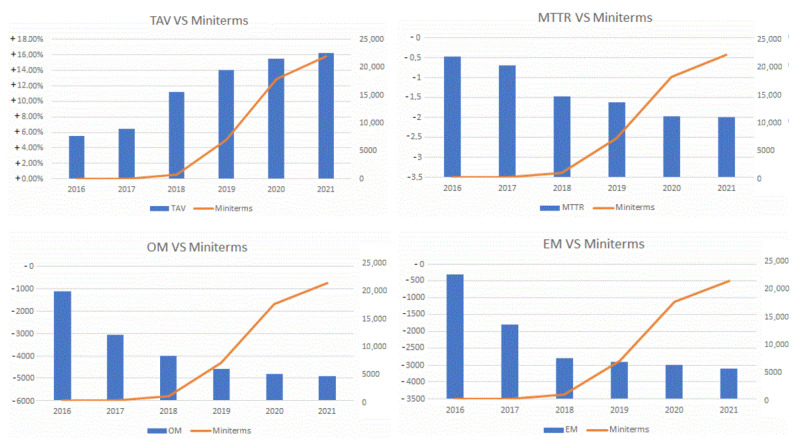
Evidence of the benefits of using mini-terms in production. NOTE: Due to industrial data protection issues, these graphs do not show the actual values of each indicator but instead they show the incremental improvement of each indicator since the initial year, 2016.

Figure 10 shows the evolution of these indicators between the years 2016–2021 and compares it with the number of mini-terms installed. Due to industrial data protection issues, these graphs do not show the actual values of each indicator but instead they show the incremental improvement of each indicator since the initial year, 2016. In this comparison, and the effects that mini-terms cause, we need to take into account the improvement of the algorithm for detecting the change points and its increased reliability over the years. With respect to TAV, it has undergone an improvement of 18% from 2016 to 2021. In the case of the MTTR, it has been reduced by 2 h. Regarding the number of work orders, these have been significantly reduced thanks to the prediction of failures, eliminating about 3000 orders/year, while working hours have also been drastically reduced by almost 4000 h/year.

## 7. Discussion

The appearance of mini-terms as virtual sensors used for predictive maintenance has become a huge development for the analysis of the behaviour of components and machines. Their main advantage is that their installation does not involve a large financial outlay and therefore Big Data can be developed for a wide variety of components and machines. Currently, as predictive maintenance requires the installation of sensors, etc., the industry often chooses critical components or machines for its installation. However, a line stop can be caused by a component as small as a switch. The mini-term allows to monitor the health of this type of components in a global way in an entire factory.

There are several limitations when using mini-terms in the industry. These include:

- The use of the industrial network for data transmission and PLCs to measure cycle times may cause certain technical limitations. These are:
  − When the number of mini-terms increases significantly, the industrial network may suffer and directly affect production, due to network saturation.
  − When the mini-term value, cycle time to be measured, is small and approaches or exceeds the PLC cycle time (Scan-Time), it generates distortions in the data and the change point can be masked within the effect generated by the Scan-Time.
- The use of the mini-terms is based on the variation of the cycle time due to deterioration. However, when the element or component has a control system, it may hide this temporary deterioration. We can take as example a welding clamp with servomotor that begins to have a mechanical deterioration. These systems have a closing speed setpoint that the control system will try to keep at all costs, hiding the deterioration from the point of view of cycle time.

## 8. Conclusions and Future Work

This article introduces a novel virtual sensor for predictive maintenance called mini-term. The advantage introduced by the mini-term is that it can be implemented in an easy and simple way in pre-installed systems since you only need to program a timer in the PLC or PC that controls the line/machine in the production line, allowing, according to the authors’ knowledge, to build industrial Big Data on predictive maintenance for the first time, which is called Miniterm 4.0. This article shows the current state of development of the Miniterm 4.0 intelligent system where, the first objective is to build a global monitoring system for technical sub-cycle times (mini-terms) that allows predicting machine/component failures in real time. After installing more than 14,000 mini-terms in the last 3 years at Ford factory in Almussafes (Valencia), evidence has been generated of how the Miniterm 4.0 system can improve production rates. Indicators such as TAV (Technical availability), Mean Time To Repair (MTTR), Mean Time Between Failure (MTBF), EM (Number of Work order (Emergency Orders/line Stop)) and OM (Labour hours in EM) have generated a very important improvement as the number of mini-terms increased and the Miniterm 4.0 system became more reliable. In particular, TAV is increased by 15%, OM is reduced in 5000 orders, MTTR is reduced in 2 h and there are produced 3000 orders less than when mini-terms did not exist.

As future work, we will intend to complement the function of the mini-term as a predictor of failures functioning as a sub-cycle time, allowing to estimate the real capacity of production lines, as well as estimating the loss due to deterioration, determining the bottleneck in dynamic, etc. A first approach to the benefits that this new capacity can bring to the Miniterm 4.0 system has been presented in [29]. 

## Data Availability

Not applicable.
